# Supplementary material for: Gaze following in Archosauria—Alligators and palaeognath birds suggest dinosaur origin of visual perspective taking
Source: Sci Adv. 2023 May 19;9(20):eadf0405. doi: 10.1126/sciadv.adf0405 (PMC10198628; doi:10.1126/sciadv.adf0405)
Supplement: Supplementary file 1 — Supplementary Text Figs. S1 to S3 Tables S1 and S2 Legends for data S1 and S2 [file sciadv.adf0405_sm.pdf]

Science Advances
AAAS

Supplementary Materials for

Gaze following in Archosauria—Alligators and palaeognath birds suggest dinosaur origin of visual perspective taking

Claudia Zeiträg *et al.*

Corresponding author: Claudia Zeiträg, claudia.zeitrag@lucs.lu.se

Sci. Adv. **9**, eadf0405 (2023)
DOI: 10.1126/sciadv.adf0405

The PDF file includes:

Supplementary Text
Figs. S1 to S3
Tables S1 and S2
Legends for data S1 and S2

Other Supplementary Material for this manuscript includes the following:

Data S1 and S2

Supplementary Text

1. Housing

1.1. Alligators

Subjects were six seven-year-old American alligators (*Alligator mississippiensis*; 2 males and 4 females) that were group-housed in an indoor facility consisting of a 42 m^2 pool area and a 26.5 m^2 land area. Subjects were tested on land by dividing the pool from the land area with opaque screens that didn't allow for visual contact with the rest of the group, but they could still hear each other.

1.2. Small birds

Small birds in this study were six adult elegant-crested tinamous (*Eudromia elegans,* 1 female and 5 males) and six adult red junglefowl (*Gallus gallus*, 1 male and 5 females), living in one social group, respectively. Elegant-crested tinamous were group-housed in an outdoor aviary during the experimental period, while red junglefowl had access to an indoor and outdoor aviary. Elegant-crested tinamous were tested in their outdoor aviary, while red junglefowl were tested indoors.

1.3. Large birds

Large birds in this study were six adult emus (*Dromaius novaehollandiae*, 3 females and 3 males) and six adult greater rheas (*Rhea americana*, 2 males and 4 females). Emus were all pair-housed, i.e., the experiments took place in three different locations. Rheas were from two different mixed-sex groups, tested in two different locations. Both species were tested in their outdoor enclosures.

Figure S1.

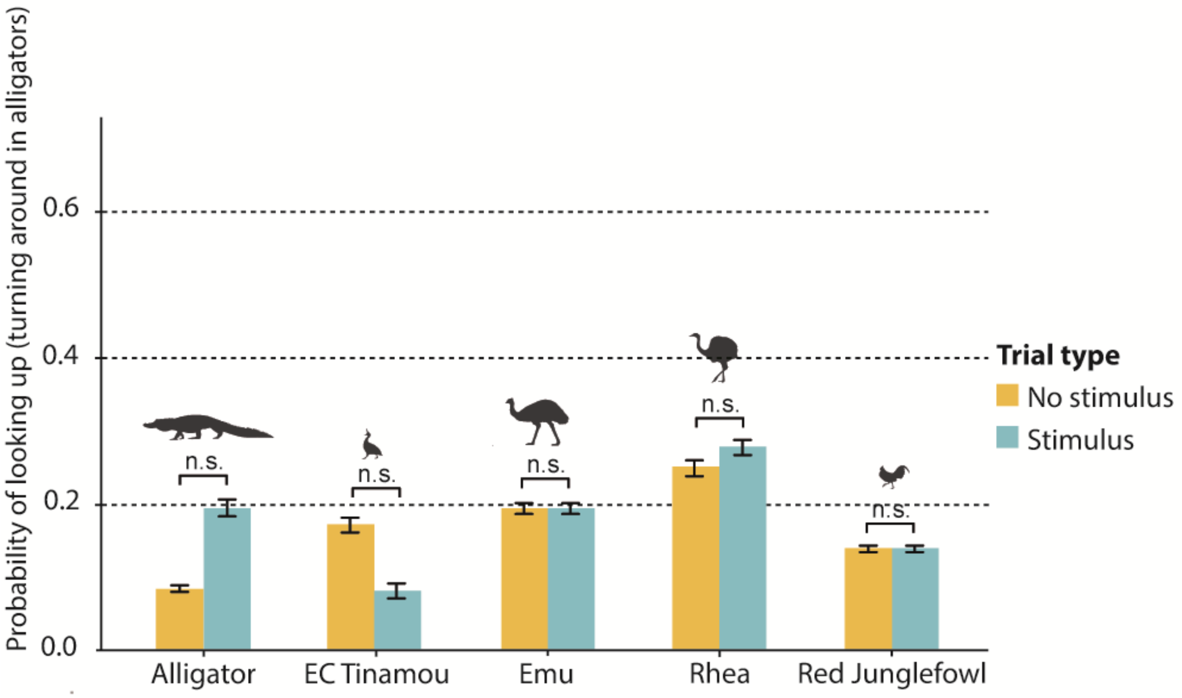

No-demonstrator condition of gaze-following into the distance: Up. Probability of looking up (turning around in alligators) in no-demonstrator condition of Experiment 1. No significant difference in the probability of looking up could be identified for the bird species in trials where the stimulus was shown compared to trials where no stimulus was shown (likelihood ratio test, $\chi^2 = 0.12$, df = 1, p = 0.73). No significant effect of species could be identified for birds (likelihood ratio test, $\chi^2 = 3.19$, df = 3, p = 0.36). For alligators, no significant difference in the probability of turning around could be identified in stimulus trials compared to no-stimulus trials of the no-demonstrator condition (likelihood ratio test, $\chi^2 = 1.86$, df = 1, p = 0.17). EC Tinamou = elegant-crested tinamou.

Figure S2.

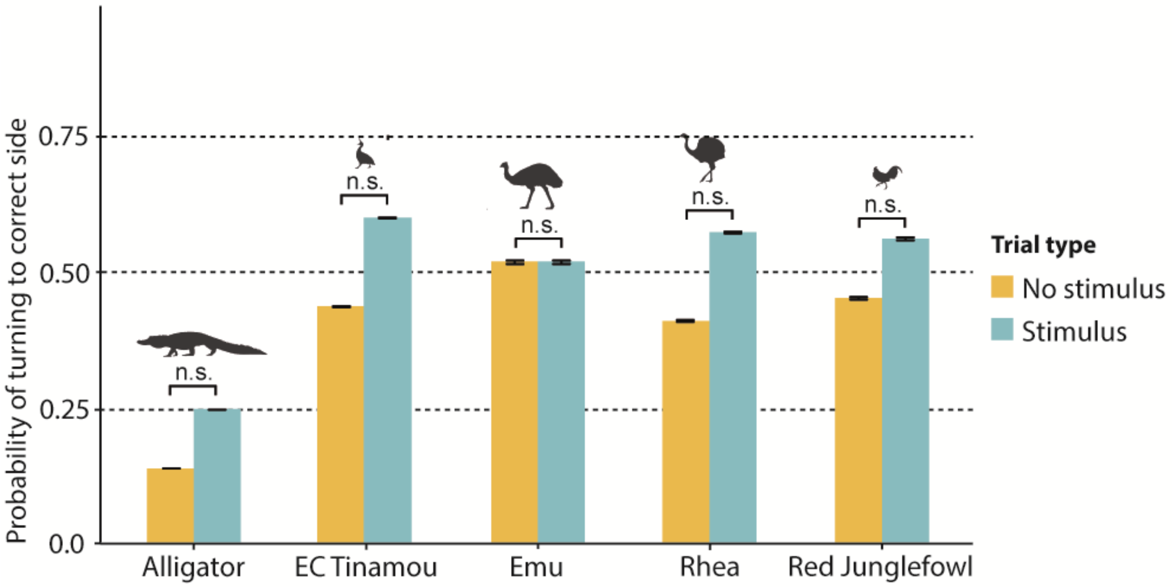

No-demonstrator condition of gaze-following into the distance: Sideways. Probability of turning to correct side in no-demonstrator condition of Experiment 2. No significant difference in the probability of turning to the correct side could be identified for birds (likelihood ratio test, $\chi^2 = 3.61$, df = 1, p = 0.05) and alligators (likelihood ratio test, $\chi^2 = 1.39$, df = 1, p = 0.24) in trials where a stimulus was shown compared to no-stimulus trials. No significant effect of species could be identified when comparing birds (likelihood ratio test, $\chi^2 = 3.19$, df = 3, p = 0.36). EC Tinamou = elegant-crested tinamou.

Figure S3.

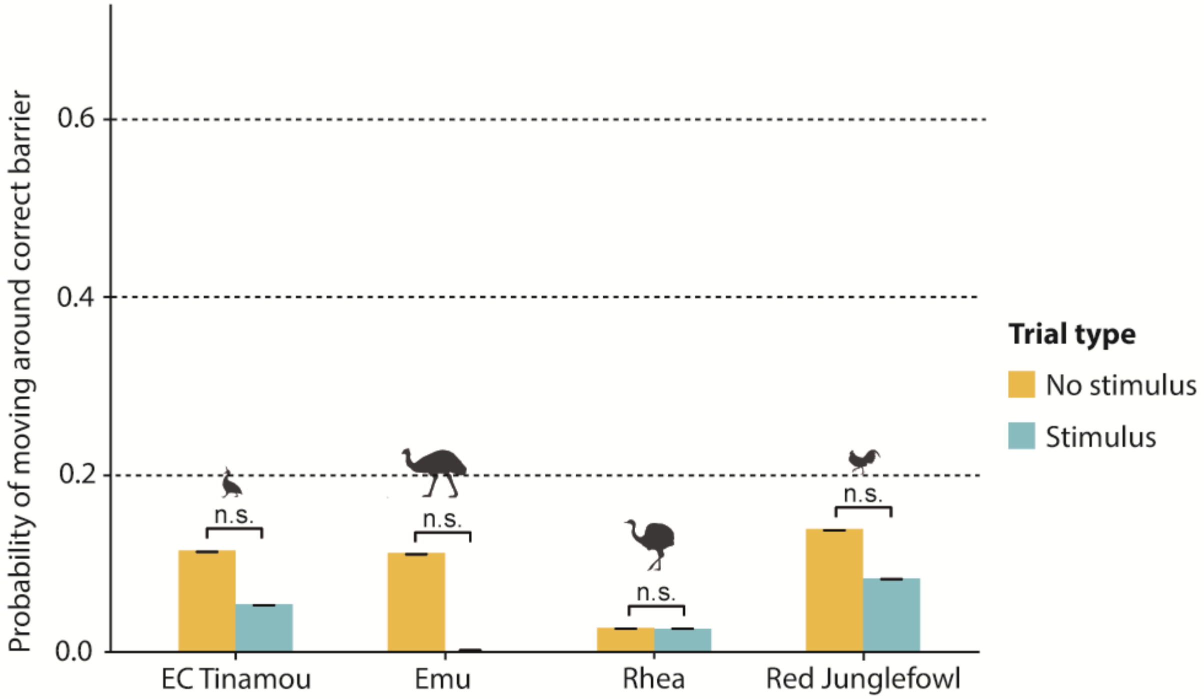

No-demonstrator condition of geometrical gaze-following. Probability of moving around correct barrier in no-demonstrator condition of bird species (Experiment 3). No significant difference in the probability of moving around the correct barrier could be identified (likelihood ratio test, $\chi^2 = 3.44$, df = 1, p=0.06). No significant effect of species could be identified (likelihood ratio test, $\chi^2 = 3.96$, df = 3, p=0.27). EC Tinamou = elegant-crested tinamou.

Table S1.

Species	Subject	Sex	Age	Role
American alligator	Toke	female	subadult	Demonstrator & Subject
American alligator	Ivar	male	subadult	Demonstrator & Subject
American alligator	Bestla	male	subadult	Subject
American alligator	Sigi	female	subadult	Subject
American alligator	Kåra	female	subadult	Subject
American alligator	Gudrun	female	subadult	Demonstrator & Subject
Emu	Snow	female	adult	Demonstrator & Subject
Emu	Tufty	male	adult	Demonstrator & Subject
Emu	Uncrowned	female	adult	Demonstrator & Subject
Emu	Crowned	male	adult	Demonstrator & Subject
Emu	Judy	female	adult	Demonstrator & Subject
Emu	Harry	male	adult	Demonstrator & Subject
Rhea	Nox	male	adult	Demonstrator & Subject
Rhea	Hamilton	male	adult	Demonstrator & Subject
Rhea	Yvette	female	adult	Subject
Rhea	Salsa	female	adult	Demonstrator & Subject
Rhea	Lucia	female	adult	Demonstrator & Subject
Rhea	Arroz	female	adult	Subject
Elegant-crested tinamou	Alicio	male	adult	Demonstrator & Subject
Elegant-crested tinamou	Sleepy Genius	male	adult	Demonstrator & Subject
Elegant-crested tinamou	Pretty Boy	male	adult	Demonstrator & Subject
Elegant-crested tinamou	New Tinamou	male	adult	Subject
Elegant-crested tinamou	Jon Snow	male	adult	Subject
Elegant-crested tinamou	Sandy	female	adult	Subject
Red junglefowl	Yellow	female	adult	Demonstrator & Subject
Red junglefowl	Pink	female	adult	Demonstrator & Subject
Red junglefowl	Red	female	adult	Subject
Red junglefowl	White	female	adult	Subject
Red junglefowl	Green	female	adult	Subject
Red junglefowl	Rooster	male	adult	Subject

Test subjects. Subjects within a species marked in the same color were housed together as a social group.

Table S2.

Model	Response variable	Distribution	Coefficient	Chisq	df	p
All species, all experiments	VCO	Binomial	Experimental Condition	18.62	2	<0.001***
			Species	15.57	4	0.0037**
			Experimental Condition*Species	8.03	8	0.43
Proportions gaze following into distance	VCO	Binomial	Experimental Condition	9.71	1	0.0022**
			Species	25.80	4	<0.001***
Experiment 1, birds, no-demonstrator condition	VCO	Binomial	Species	3.19	3	0.36
			Test Condition	0.12	1	0.73
Experiment 1, birds, demonstrator-condition	VCO	Binomial	Species	4.47	3	0.21
			Test Condition	16.33	1	<0.001***
			Species*Test Condition	4.40	3	0.22
Experiment 1, alligators, no-demonstrator condition	TA	Binomial	Test Condition	1.86	1	0.17
Experiment 1, alligators, demonstrator condition	TA	Binomial	Test Condition	5.77	1	0.01*
Experiment 2, birds, no-demonstrator condition	VCO	Binomial	Test Condition	3.61	1	0.05

Experiment 2, birds, demonstrator condition	VCO	Binomial	Species	9.26	3	0.03*
			Test Condition	14.73	1	<0.001***
			Species*Test Condition	5.67	3	0.13
Experiment 2, alligators, no-demonstrator condition	VCO	Binomial	Test Condition	1.39	1	0.24
Experiment 2, alligators, demonstrator condition	VCO	Binomial	Test Condition	4.09	1	0.04*
Experiment 3, birds, no-demonstrator condition	VCO	Binomial	Species	3.96	3	0.27
			Test Condition	3.44	1	0.06
Experiment 3, birds, demonstrator condition	VCO	Binomial	Species	4.88	3	0.18
			Test Condition	33.74	1	<0.001***
Checking back birds	CB	Binomial	Experimental Condition	4.65	2	0.098
			Species	9.72	3	0.021*
			Experimental Condition*Species	13.75	6	0.033*

Results of likelihood ratio test performed on the final Generalized Linear Mixed Models (lowest AIC). VCO = visual co-orientation; TA = turning around; CB = checking back.

Data S1. (separate file)
Data sheet.

Data S2. (separate file)
Code as R Script.
